# Host selection pattern and flavivirus screening of mosquitoes in a disturbed Colombian rainforest

**DOI:** 10.1038/s41598-021-98076-8

**Published:** 2021-09-20

**Authors:** Juliana Hoyos, María Cristina Carrasquilla, Cielo León, Joel M. Montgomery, Stephanie J. Salyer, Nicholas Komar, Camila González

**Affiliations:** 1 Department of Biological Sciences, Center for Research in Tropical Microbiology and Parasitology (CIMPAT), University of Los Andes, Bogotá, Colombia; 2 Viral Special Pathogens Branch, Division of High Consequence Pathogens and Pathology, National Center for Emerging and Zoonotic Infectious Diseases, U.S. Centers for Disease Control and Prevention, Atlanta, Georgia; 3 Global Epidemiology, Laboratory, and Surveillance Branch, Division of Global Health Protection, Center for Global Health, U.S. Centers for Disease Control and Prevention, Atlanta, Georgia; 4 Arbovirus Diseases Branch, Division of Vector-Borne Diseases, National Center for Emerging and Zoonotic Infectious Diseases, U.S. Centers for Disease Control and Prevention, Ft. Collins, CO USA

**Keywords:** Ecological networks, Entomology, Community ecology

## Abstract

Studies on the feeding behavior of hematophagous insects, particularly those of medical importance, are relevant for tracking possible pathogen transmission routes and identifying biases in the choice of vertebrates. We evaluated host selection of blood-feeding mosquitoes in a disturbed forest in the Magdalena Medio valley in Colombia from March 2017 to April 2018, after the introduction of Zika virus to the Americas from the 2015–2016 outbreak. We estimated vertebrate diversity and collected blood-engorged female mosquitoes. Genomic DNA/RNA was extracted from the mosquito’s abdomen for vertebrate host identification and pathogen detection. We performed conventional PCR and sequencing, using universal primers targeting vertebrate regions of the eukaryotic mitochondrial genome to determine bloodmeal host. Additionally, we tested for the presence of flaviviruses in all mosquito samples with RT-PCR. Based on the identity and quantity of detected bloodmeals, we performed mosquito-vertebrate interaction network analysis and estimated topology metrics. In total, we collected 292 engorged female mosquitoes representing 20 different species. Bloodmeal analyses identified 26 vertebrate species, the majority of which were mammals (N = 16; 61.5%). No flaviviruses of medical importance were detected from the samples. Although feeding patterns varied, network analyses showed a high degree of specialization by mosquitoes and revealed ecological and phylogenetic relationships among the host community. We conclude that host selection or preference by mosquitoes is species specific.

## Introduction

Transmission of mosquito-borne pathogens depends on the vertebrate host selection of mosquitoes when seeking bloodmeals for their egg development. In ecosystems with anthropogenic disturbances, defining vector feeding patterns (specialist vs opportunistic), is key to assessing potential changes in pathogen transmission cycles and the risk of pathogen spillover^1–4^. Some mosquito species are generalists and often show opportunistic feeding behavior such as those in the *Culex* genus, which feed on a diverse range of species^5,6^. Others are specialists, such as *Aedes aegypti* who feeds primarily on humans^7^. In the sylvatic-urban interface, humans, mosquitoes, and wild animals co-occur, increasing the risk of zoonotic disease spillover to humans^8^. Additionally, habitat disturbance by humans may facilitate the dispersion of anthropophilic mosquito species, changing vector-host interactions and driving increased contact between human-biting mosquitoes and wild reservoirs of zoonotic pathogens^9^. Opportunistic mosquito species can act as bridge vectors between wild and urban transmission cycles^10^, increasing the risk of spillover from sylvatic to urban environments and vice versa^11^. Thus, understanding the interactions among mosquitoes and the vertebrates on which they feed is of great importance^1–3^. Mosquito-borne viruses associated with sylvatic and urban transmission cycles, constitute an important challenge for the design of prevention and control strategies because they usually involve a diversity of vector and reservoir species in complex multi-pathogen multi-host dynamics^11,12^. For example, West Nile virus (WNV) can replicate in a great variety of Culicine vectors and vertebrate hosts and can affect hundreds of species from various classes^13,14^.

In Colombia, many pathogenic arboviruses are enzootic/endemic with many mosquito species potentially involved as vectors^15–17^. However, data from ecological studies on host selection by those mosquitoes of medical importance are scarce^6^. This study aims to evaluate the patterns of host selection by mosquitoes, in relation to the availability of vertebrates using network analysis, and to detect flavivirus infection in mosquitoes.

## Results

### Mosquito diversity

**Figure 1:**
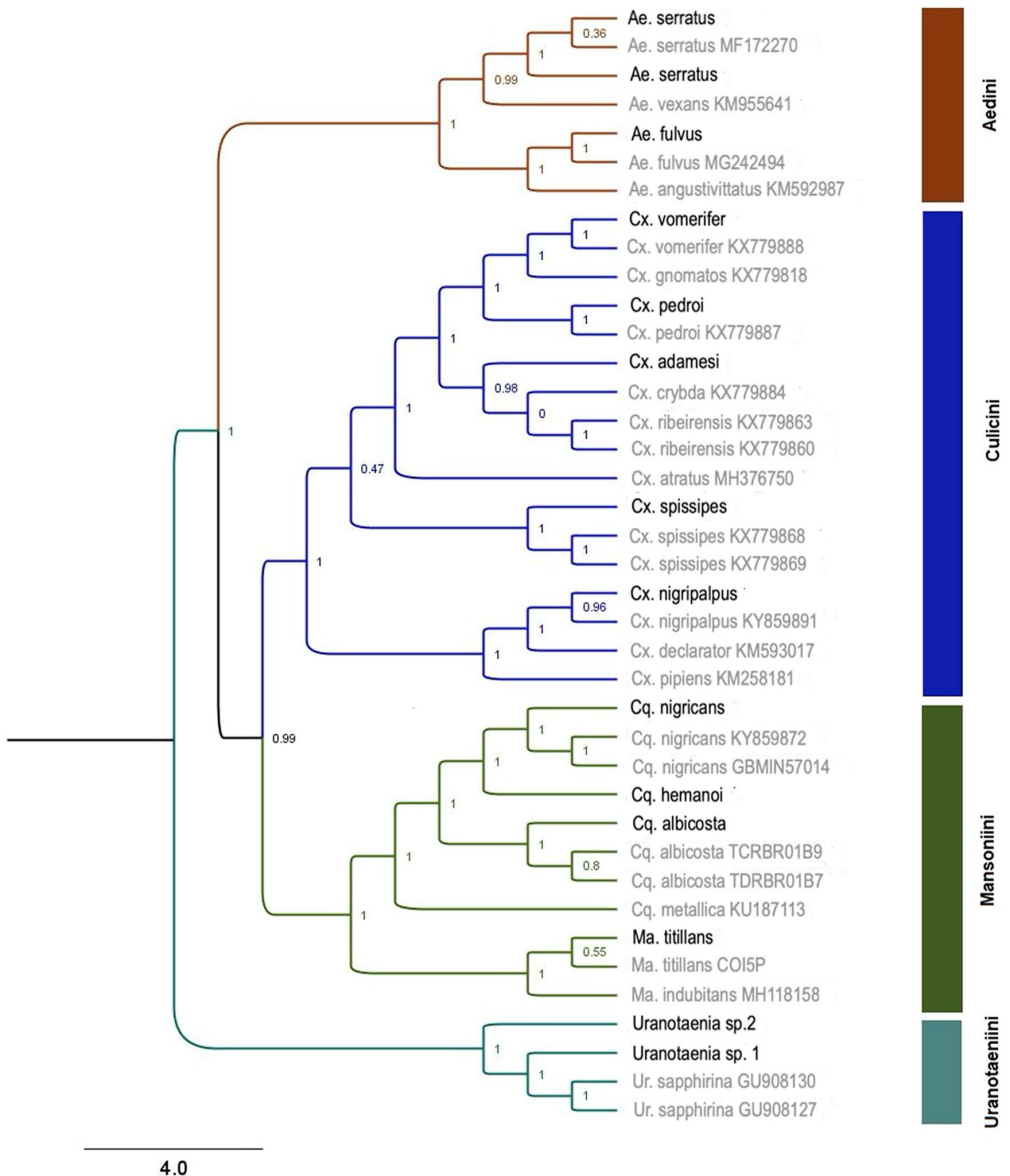
Maximum likelihood phylogenetic reconstruction based on COI sequences deposited in GenBank and our original data for the species of the family Culicidae: The reference sequences are gray, and the sequences generated in this work are black. SH‐like branch support is depicted above or in front of the corresponding nodes. The aLRT branch support is shown with ≤ two decimals. Branches are highlighted by Tribe level.

From six field campaigns,we collected 292 blood-engorged female mosquitoes. Twenty mosquito species were identified by morphology representing eight genera. By analyzing the obtained Cytochrome c Oxidase Subunit I (COI) sequences, we confirmed the identity of 11 of these species (Table 1). The most abundant species was *Aedes fulvus* (15.8%), and the most diverse genus collected was *Culex*, with seven species identified, followed by *Uranotaenia* with four species. Genetic distances, as determined by the Kimura-2-parameter (K2P) for intraspecies values, were under 2.4% for all species. The maximum likelihood (ML) tree, built using the K2P distances between species, is shown in Fig. 1. Confirmation of species identity through barcodes was achieved for nine individuals with genetic identities to reference sequences above 97.6%. Reference sequences for *Culex adamesi* were not available in any published database, so we contributed the first sequences for this species. Also, species belonging to the genus *Uranotaenia* could not be morphologically identified and our barcodes showed only 92% of homology with *Uranotaenia sapphirina*, the closest species in the barcode database.

**Table 1:** Diversity and abundance of blood-engorged female mosquitoes collected from March 2017 to April 2018 using four sampling methods in forest fragments in San Juan Carare, Colombia. The abundance of each species is calculated relative to the total number of engorged females collected (N = 292). The number of bloodmeals identified includes the result of Cyt-b and COI amplifications (N = 112). *denotes species confirmed using DNA barcodes. Mammals (M), Reptiles (R), Birds (B) and Amphibians (A).

Species	Engorged females collected	Females with identified bloodmeals	Vertebrate species type detected in bloodmeals
Aedes fulvus*	46	33	M = 32, R = 1
Aedes serratus*	39	15	M = 13, B = 2
Culex pedroi*	35	12	M = 4, R = 1, B = 7
Mansonia titillans*	30	4	M = 1, B = 3
Culex spissipes*	28	5	M = 1, R = 2, B = 2
Coquillettidia albicosta*	22	3	M = 3
Culex nigripalpus*	21	11	M = 4, B = 7
*Psorophora albipes*	20	13	M = 13
Coquillettidia nigricans*	16	2	M = 2
Culex adamesi*	10	5	M = 3, B = 2
Culex vomerifer*	6	0	
*Uranotaenia *sp. 1*	5	5	A = 5
*Anopheles neomaculipalpus*	2	1	M = 1
*Anopheles triannulatus*	2	1	M = 1
*Culex *sp. 1	2	1	A = 1
*Culex *sp. 2	2	0	
*Limatus durhami*	2	1	M = 1
*Uranotaenia *sp. 2*	2	0	
*Uranotaenia *sp. 3	1	0	
*Uranotaenia *sp. 4	1	0

### Vertebrate diversity

During fieldwork, we recorded 90 bird species belonging to 23 families using mist net collections and visual observations in three habitat types: pasture, forest understory, and river edge. Avian families with the greatest observed local diversity were Tyrannidae (13 species) followed by Ardeidae (egrets), Psittacidae (parrots), Icteridae (New World blackbirds, cowbirds, orioles), Picidae (woodpeckers) and Thraupidae (tanagers) with five species each. The bird species most frequently detected were Smooth-billed Ani (*Crotophaga ani*)*,* Great Egret (*Ardea alba*)*,* Ruddy Ground-Dove (*Columbina talpacoti*)*,* Bicolored Wren (*Camphylorynchus griseus*) and Bare-faced Ibis (*Phimosus infuscatus*). Mammal populations were assessed based on captures of 164 individuals belonging to 21 species of nine families. Chiroptera was the order with highest richness with 15 species, followed by Primates (four species), Didelphimorphia (one species) and Rodentia (one species). Additionally, nine species of reptiles and eight of amphibians were recorded (Supplementary Table S1).

### Bloodmeal analysis

**Figure 2:**
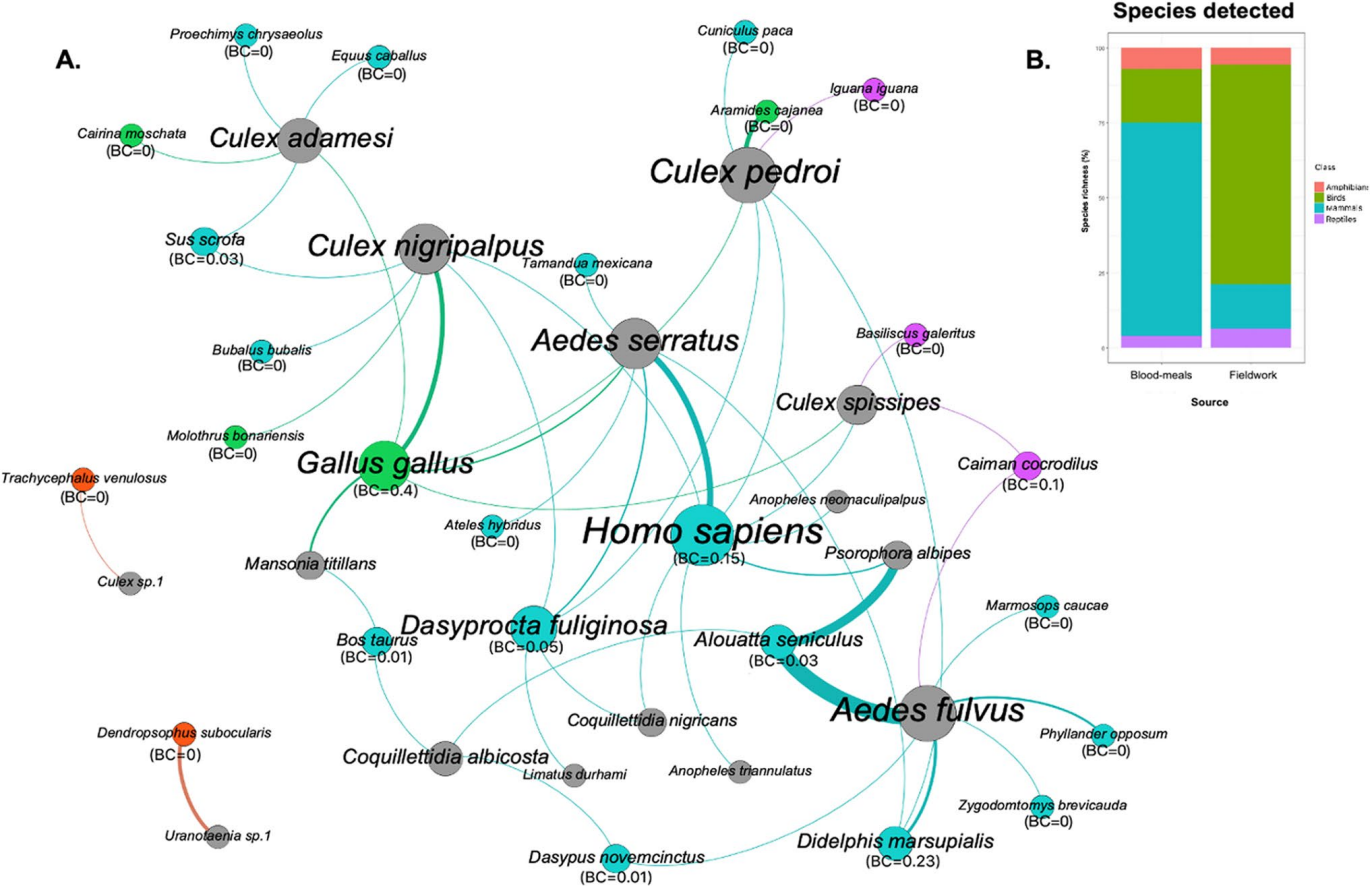
Vertebrate sources detected. (**A**) Bipartite graph of the interactions inferred from mosquito bloodmeal identification. Host species serving as blood sources are linked by shared mosquito species. Mosquito are represented by nodes in grey color, blue for mammal species, green for bird species, purple for reptiles and orange for amphibians. Species node size is relative to the number of different interactions for the species. Sample size is represented by the thickness of the link. For each vertebrate species the value of betweenness centrality (BC) was added. (**B**) Proportional contribution by each vertebrate class in the total species richness observed in the study. The left bar represents the percentages of species identified among vertebrate bloodmeals imbibed by mosquitoes. The right bar represents the percentages of species identified through observational surveys.

From the blood-engorged female mosquitoes, we amplified DNA sequences in 154 of the 292 samples analyzed, using the primers targeted to vertebrate COI (33.7% of the samples) and Cyt-b (66.3%) genes. From these 154 amplicons, 112 derived sequences matched with a homology value above our defined threshold. Vertebrate species identified included 17 mammals, four birds, three reptiles, and two amphibians. From these, 20 were sylvatic and five domestic, besides human blood meals. From the total number of sequences, 21.9% did not have enough resolution for specific identification, probably as a result of mixed bloodmeals in the sample. Vertebrate bloodmeals most frequently derived from the howler monkey (*Alouatta seniculus*, 27.7%), which was fed upon by *Aedes fulvus* and *Psorophora albipes*. *Aedes fulvus* was the mosquito species with the largest number of vertebrate species (N = 7, 26%) detected in its diet. A bipartite network constructed from bloodmeal identifications shows the total number of vertebrate species belonging to each class (Fig. 2a). Mammals provided the highest proportion of bloodmeals (Fig. 2b) and eight species from the orders Pilosa, Didelphimorphia, Rodentia and Cingulata were only detected with this method. Domestic animals were less common sources for bloodmeals, and included chicken, pig, horse, water buffalo and duck. These species were mainly found in the diet of *Cx. nigripalpus*, *Cx. adamesi* and *Mansonia titillans*. Human blood was detected in eight species: *Ae. serratus*, *An. neomaculipalpus, An. triannulatus, Psorophora albipes, Coquillettidia venezuelensis, Cx. pedroi, Cx. spissipes* and *Cx. nigripalpus*. Bird species were only detected in *Culex* and *Mansonia* mosquitoes. Overall, species belonging to the class Mammalia were more often used by the mosquitoes found in the area, followed by birds, reptile and amphibian blood in this decreasing order (Fig. 2b).

### Flavivirus detection

Flavivirus amplification was detected in 17 pools (24.3%), and 25 individuals (10%) within the pools were positive for the detection of the flavivirus NS5 gene. Retesting these 25 RNA samples for detection of the Zika virus envelope gene yielded negative results. The cDNA obtained in each reaction was revealed by agarose gel electrophoresis, but we did not obtain unique bands or viral sequences through Sanger sequencing in any of the samples. Sequencing the amplicons of the NS5 targets failed to detect known flaviviral sequences.

### Network analysis

**Figure 3:**
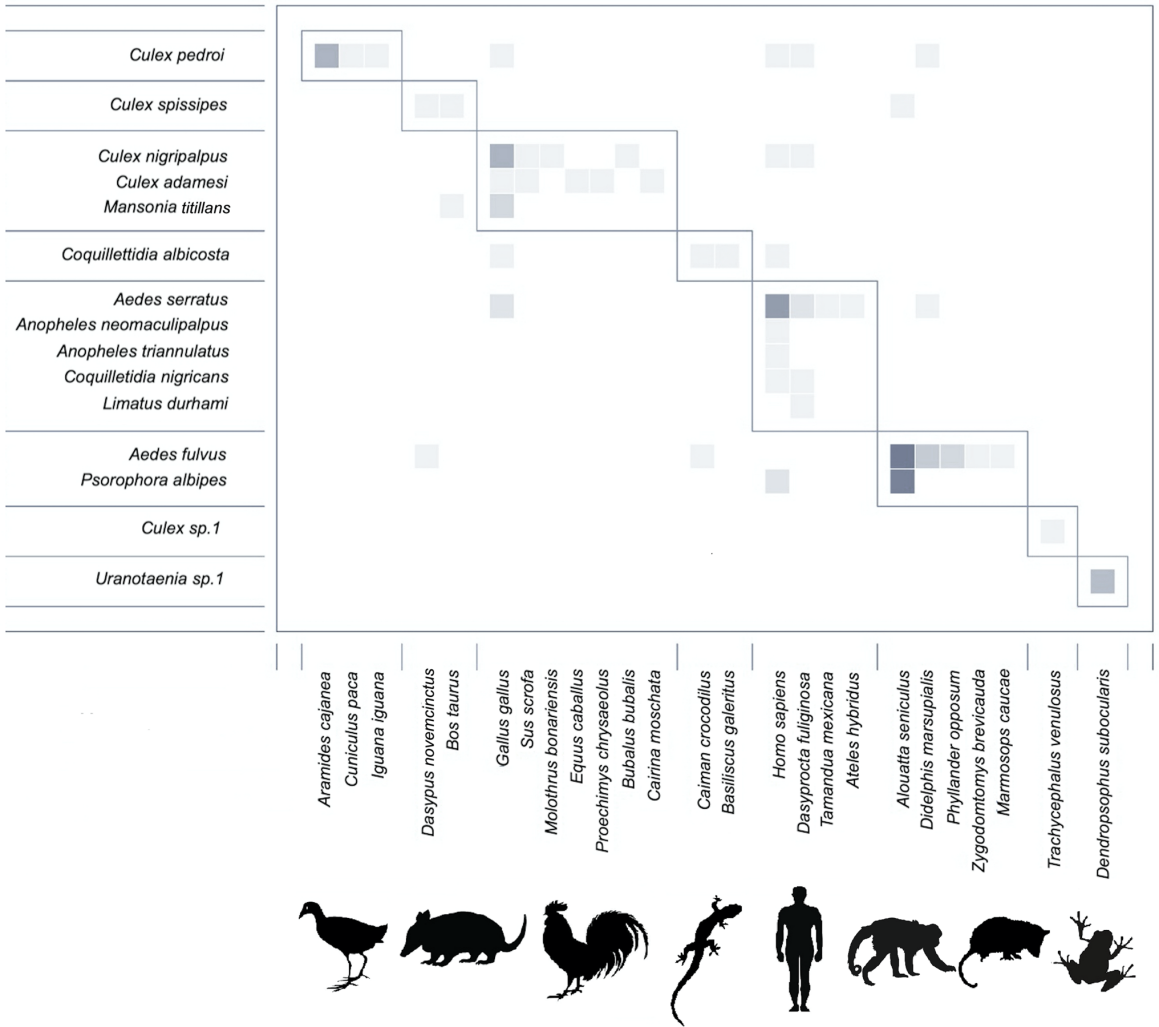
Matrix of interaction emphasizing modular relationships between vertebrate bloodmeal sources and adult female mosquitoes that fed upon them. Modularity (Q) was measured by QuanBiMo (with 50 iterations, Q_max_ = 0.59). Boxes delineate the eight modules. Darker squares indicate a greater number of interactions detected, and lighter colors fewer interactions detected. The analysis detected eight modules or subsets of mosquito species interacting more frequently with specific subsets of vertebrates.

The mosquito-vertebrate network comprised 41 different species interactions (Fig. 2a). Modularity was high (Q = 0.59, z = 13.4, *p* < 0.05), indicating significant specialization of mosquito choice of vertebrates utilized for bloodmeals. We obtained eight modules after 50 iterations (Fig. 3). Five of the modules were associated with a single mosquito species, meaning they showed a very different behavior compared to other species. The remaining modules grouped two, three or five species of mosquitoes. Regarding vertebrates, interestingly, we found one host module composed of domestic and synanthropic species, three exclusively of mammalian species, one of only reptile species and two only of amphibians. All species of marsupials (*D. marsupialis, P. opposum,* and *M. caucae*) were in the same module with the primate *Alouatta seniculus* and the rodent *Zygodontomys brevicauda*. Overall, vertebrates with similar traits in terms of habitat use, were grouped together.

The specialization index at the community level (H2′) had a value of 0.55, indicating aggregated patterns of mosquito feeding behavior and preferences for certain vertebrates. In addition, the analysis of nestedness confirmed that mosquito-feeding behavior is not significantly nested (NODF = 6.84, *p* = 0.62). At the species level, the degree of mosquito specialization varied among species when comparing the d and COV indexes. The species *Culex* sp. 1 and *Uranotaenia* sp. 1 had the highest values for both indexes (d = 1, COV = 1) suggesting those were the species with the most exclusive interaction types and narrow host range. *Anopheles neomaculipalpus* and *An. triannulatus* had a low value of index d = 0.21, but high value for the COV index = 1 (Table 2) as human blood was the only bloodmeal detected on these mosquitoes, but also in other species. *Aedes fulvus, Ae. serratus Cx. adamesi, Cx. nigripalpus,* and *Cx. pedroi* had the highest values for “species strength”, denoting greater diversity in the detected interactions. Lastly, the value of betweenness centrality (BC) for host species varied from 0 to 0.39 (Fig. 2a).

**Table 2:** Index values of specialization by mosquito species recognized in San Juan, Carare, Colombia. The Specialization index (d) and the Coefficient of interaction variation (COV) are estimated with scaled values between 0 to 1.

Species	d	COV	Species strength
*Aedes fulvus*	0.62	0.66	5.28
*Aedes serratus*	0.49	0.55	3.14
*Anopheles neomaculipalpus*	0.21	1	0.06
*Anopheles triannulatus*	0.21	1	0.06
*Coquillettidia albicosta*	0.45	0.55	1.02
*Coquillettidia nigricans*	0.27	0.69	0.22
*Culex nigripalpus*	0.54	0.56	3.15
*Culex adamesi*	0.74	0.41	3.57
*Culex* sp. 1	1	1	1
*Culex pedroi*	0.61	0.51	3.46
*Culex spissipes*	0.45	0.47	1.63
*Limatus durhami*	0.49	1	0.17
*Mansonia titillans*	0.52	0.78	0.71
*Psorophora albipes*	0.35	0.86	0.48
*Uranotaenia* sp. 1	1	1	1

## Discussion

Using morphology and molecular analyses, we could identify 11 mosquito species previously involved in arbovirus transmission, out of 20 species of mosquitoes collected^15–17^, with the greatest number of species belonging to the *Culex* genus (six species). Species in the genus *Culex*, especially those belonging to the *Melanoconion* subgenus, are recognized as a highly diverse group with great taxonomic difficulties^18^, due to morphological similarity and the lack of recent taxonomic revisions of the *Melanoconion* subgenus^19,20^. The use of mitochondrial COI gene has become a great help in delimiting species within groups displaying ambiguous morphological traits^21^; we contributed the COI sequences for 14 mosquito species from an unsampled ecoregion. The efficiency of species identification was supported by the barcoding gap analysis, which implies that if a gap exists, a cut-off value should be defined allowing a clear distinction between the interspecific and intraspecific distances. The K2P intraspecific and interspecific values obtained here are similar to values found in other studies that include related species^22^.

We identified 26 species of vertebrates among 154 mosquito bloodmeals, and found that only 10 of these species were observed during fieldwork. Four species *P. opposum, M. caucae, D. novemcinctus,* and *Z. brevicauda,* were only detected through bloodmeal analyses and were expected at the study site based on available distribution records^23^. We only placed vertebrate traps at the ground level, so we missed arboreal mammals during sampling; future studies should include trapping at different levels in the forest to better characterize the vertebrate community^24^.

Human DNA was detected in 6.9% of the identified bloodmeal sequences in eight mosquito species, some previously known to feed on humans: *Ae. serratus*, *Ps. albipes*, *Cx. nigripalpus*, *An. triannulatus* and *Cq. nigricans*^25–29^. Studies in villages located in the Brazilian amazon forest found that *Ae. fulvus* fed on humans and peridomestic animals such as dogs, pigs, and cows^25^, while Silva et al*.*^26^ found a greater tendency to ornithophily in this species. These data contrast our findings, considering that despite the abundance of samples obtained, we did not find evidence of *Ae. fulvus* feeding on humans or birds, and the selection of hosts for this species was dominated almost entirely by sylvatic mammalian species. *Culex nigripalpus* has been recognized as a species with broad host selection in laboratory-controlled experiments^28^, and in our study, it also showed a wide-ranging feeding behavior, mainly related to domestic animals. Similar to our findings, Mitchell et al.^30^ identified *Mansonia titillans* frequently feeding on domestic species including Phasianidae birds and Bovidae. Preference of mosquitoes for vertebrates in nature can be modulated by many variables, including but not limited to relative abundance of vertebrate hosts, reproductive cycles, and host defensive behavior^31^. These ecological factors are difficult to monitor, limiting the understanding of innate behaviors of the vector^32–35^. One very specific behavior is that of the genus *Uranotaenia* which has been associated exclusively with ectothermic animals. We found *Uranotaenia* sp. 1 feeding on the amphibian *Dendropsophus subocularis*. The species *U. lowii* was one of the first mosquitoes in the Neotropics to show attraction towards the sound of an amphibian host^36^. In our study some *Culex* species were also attracted to ectothermic animals (frogs) as food source.

For most of the mosquito species in which human blood was identified, except *An. triannulatus* and *An. neomaculipalpus*, more than one host was found. Factors favoring humans as food sources and their epidemiological consequences merit further investigation. Frequent contact with a host leads to a strong vector-vertebrate interaction that improves the transmission of pathogens^37^. Mosquitoes with low specialization have special public health relevance, since they can disperse through a diverse range of ecosystems, becoming more likely to get infected with new pathogens, and raising the risk of spillover^10^. Conversely, a greater diversity of interacting vertebrate species favors a dilution effect, and this allows prevalence rates to remain low^38^.

Although the feeding patterns found in our study are varied, according to our analysis (significant modular topology Q) we conclude that they don’t occur randomly, and there is a preference of mosquitoes for certain vertebrates. Among ecological networks, values of modularity and specialization tend to be higher in antagonistic-relationship networks if compared to other types of interactions such as mutualism^39,40^. The species involved in such antagonistic interactions tend to form enclosed modules within the interaction matrix^41^. This marked compartmentalization within antagonistic networks (species grouped in discrete modules) occurs as a consequence of a series of pressures (evolutive, physiological, etc.), establishing restrictions over their interactions^40^. In a parasite-host modular network, the hosts of the same module are used by the same group of parasites, in our case by the same group of mosquitoes^42^. In our study site, for example, *Aedes* and *Anopheles* feed on a variety of sylvatic mammalian species, while only *Uranotaenia* and *Culex* fed on amphibians.

In modular networks, sub-communities defined by such modules are loosely connected to one another. Within this network topology, some nodes act as bridges connecting the different communities, facilitating perturbations to spread throughout the entire network^43^. The value of Betweeness Centrality calculated for each vertebrate species can be used to identify individuals that ‘bridge’ distant groups and may thus play an important role in spreading parasites among network sub-communities^44^. Among the detected vertebrates, chickens, common opossums and humans showed the highest values of betweenness centrality, denoting their potential role as bridge species between sub-communities. For instance the common opossum is a synanthropic species with home range on the urban-sylvatic interface, and host for a wide range of vector-borne pathogens^45,46^. The role of humans must also be considered for pathogen transmission from urban to sylvatic cycles, as has been shown in South America for imported pathogens such as YFV and dengue virus^1^.

Regarding flavivirus detection, we expected to find infection since the mosquito species collected are known to be involved in human arbovirus transmission in the Neotropics. For instance, *Ae. serratus*^47^ and *Mansonia titillans*^48^ have been implicated as potential secondary vectors of yellow fever in Brazil and have been found susceptible and potentially involved in Mayaro virus transmission. Also, *Ae. fulvus* and *Cq. nigricans* have been found infected with Madariaga virus^16,49^. However from the 25 samples that tested positive in the initial PCR screen for flavivirus, we didn’t detect any known virus. Some of the “false positives” may have been a combination of flavivirus targets combined with false targets in the blood component. Mixed amplicons are generally uninterpretable by Sanger sequencing, and require either cloning or next generation sequencing methods to resolve the resulting sequence ambiguities.

In conclusion, our work provides an important contribution in the field of disease ecology by identifying mosquito-vertebrate interactions including species with medical importance and establishing well-defined patterns of host selection by Colombian mosquitoes.

## Methods

### Study site

**Figure 4:**
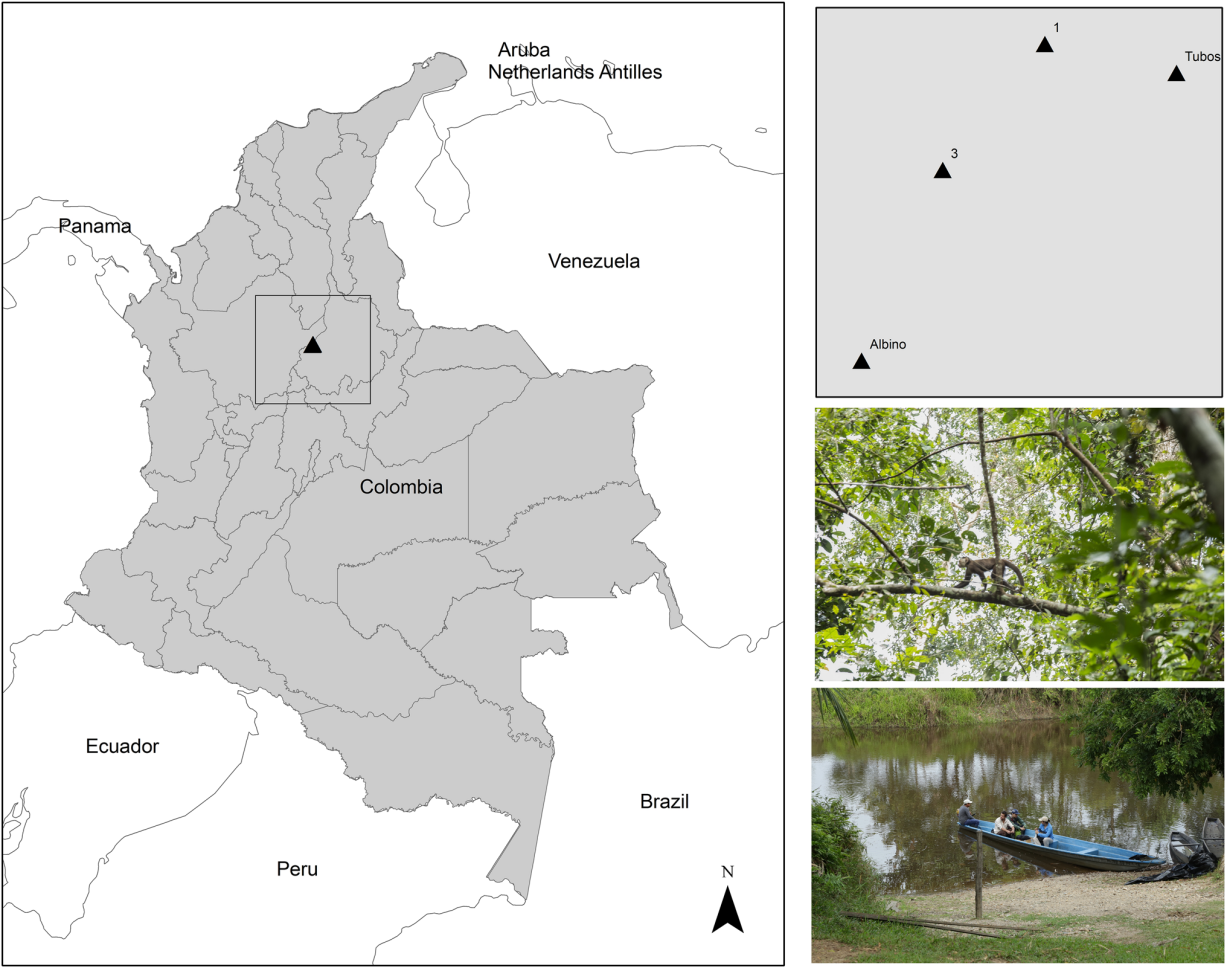
Map of the study site in San Juan Carare, Santander, Colombia, showing the location of transects within forest fragments and the nearest human settlement. The four tiangles show the distribution of the fragments sampled. The map was generated using ArcGis 10.7.1. (https://desktop.arcgis.com/en/arcmap/) Copyright 1995–2018 Esri. All rights reserved. Published in the United States of America.

Our sampling site was the locality of San Juan, Carare, in the department of Santander, Colombia (06°43′N, 74°09 ′W; 170 to 210 m.a.s.l) (Fig. 4). The area is a flooded tropical rainforest located between the central and eastern Andes, in the ecoregion of Magdalena Medio River Valley. The region is known to support alphavirus and flavivirus transmission with high mosquito diversity^50^ and the presence of sylvatic fauna, particularly primates^51^. This area, as most of the Magdalena river valley, has been deforested by agriculture and livestock activities, generating a matrix of primary humid forest fragments with shrub-like areas mixed with crops^52^. We sampled during six campaigns at this site between March 2017 and April 2018.

### Mosquito and vertebrate collection and identification

We collected mosquitoes by establishing four transects, each with three CDC miniature light traps, three BG-Sentinel traps baited with BG-lure, and three resting traps, separated by 10 m. All traps remained active from 18:00 h to 6:00 h during five days in each campaign. Additionally, Prokopak^®^ aspirators were used at the undergrowth level during two consecutive hours in the evening, two days in each of the six campaigns for a total sampling effort of 24 h of aspiration. After collection, we sacrificed the insects using ethyl acetate and sorted them, keeping all the Culicidae. We stored the mosquitoes in 1.5-mL Eppendorf® tubes by trap-night collection in liquid nitrogen, until transport to the Research Center in Tropical Microbiology and Parasitology (CIMPAT for its acronym in Spanish) at the Universidad de Los Andes. In the laboratory, we selected female mosquitoes with blood-engorged abdomens for molecular analyses, and the remaining specimens were kept for a separate analysis. We identified mosquito species according to external morphology using available taxonomic keys^53,54^, and species identity confirmation was accomplished by DNA barcoding. High Pure PCR Template Preparation Kit (Roche) was used for extraction of nucleic acid following the manufacturer's protocol. The DNA barcode was amplified from a 658- bp region of the mitochondrial COI gene^55^. The PCR mixture was prepared to a final volume of 25 μL which contained 12.5 μL of 2 × GoTaq Green Master Mix (Promega), 10 μM of primers LCO1490 and HCO2198 and 5 μL of DNA. To determine the band size, amplification products were visualized on 2% agarose gels and stained with Sybr Safe (Invitrogen). The amplification products were sequenced bi-directionally at Gencore, Universidad de Los Andes. Raw sequences were assembled using DNAbaser and aligned with reference sequences from GenBank and the Barcode of Life database (BOLD). Pairwise nucleotide sequence divergence was estimated among all sequences using the K2P model implemented in MEGA X software. The platform ABGD software (Automatic Barcode Gap Discovery) was used to find values of intra and interspecific distance (barcode gap) for species delimitation^56^. Phylogenetic reconstruction was performed with PhyML 3.0^57^ using the GTR + I + G nucleotide substitution models suggested by the SMART model selection and branch support was evaluated with the aLRT.

Additionally, to have an estimate of vertebrate species richness and relate it to blood sources found in mosquitoes, three transects each with 20 Sherman traps and 10 Tomahawk traps were set, for a total capture effort of 90 traps/night. Additionally, bats were surveyed using four mist nets, from 6:00 pm to 10:00 pm during eight consecutive nights during each of the six field trips to the study region. An additional field trip of five days for bird survey was conducted (June, 2017); during this survey, we conducted ten point counts for 20 min each, distanced by 250 m apart, during the peaks of activity (close to sunrise and sunset).

### Blood-meal analysis

To detect vertebrate blood sources, we removed the abdomen from each engorged female mosquito with blood content greater than 60% and extracted nucleic acid with DNA/RNA MiniPrep Viral kit (Zymo, Inc.) following the manufacturer's instructions. We used conventional PCR with universal primers for the amplification of a region of the mitochondrial cytochrome b (Cytb) gene of birds and mammals^58,59^. For samples that did not amplify, we used primers for amplifying a region of the mitochondrial COI gene^2^. PCR was performed in a reaction containing 12.5 μL of 2 × GoTaq Green Master Mix® (Promega), 5 μM of each primer and 2.5 μL of DNA. The amplification products were visualized on agarose gels, and positive samples were sequenced by capillary electrophoresis (the Sanger method) using an ABI-3500 genetic analyzer from Life Technologies (3500 Genetic Analyzer) at the Gencore Laboratory. We edited the raw sequences using the DNA Baser software (Heracle BioSoft SRL) and compared with sequences available in the GenBank (National Biotechnology Information Center) and Barcode of Life Data System (BOLD) databases through the BLAST platform using the Blastn algorithms, and when possible, confirmation with Blastx. Samples were identified accepting homology values ​​higher than 96%.

### Flavivirus detection

To detect infected females we first tested pools of RNA derived from four individual mosquito abdomens (2 μl each), and when a positive pool was found, the individual RNA samples were retested individually (8 μl). This one-step RT-PCR assay utilized the Quantitect SYBR Green RT-PCR kit (Qiagen) following manufacturer’s recommended protocol with primers PF1S and PF2R-bis, which recognize a 260 bp target in the NS5 gene of known flaviviruses^60^. Nucleic acid was extracted manually using the DNA/RNA Viral MiniPrep kit (Zymo, Inc.). Amplification of a viral target sequence was considered successful when the fluorescence signal indicated a peak with a melting temperature between 75 and 85 °C. Prevalence was calculated as the number of positive samples over the number of processed samples (%). Flavivirus-positive females were evaluated for Zika virus infection in a second real-time RT-PCR following the protocol previously described^61^. Each reaction corresponded to a total volume of 50 µL containing 18.2 µL RNAase-free water, 25.0 µL Quantitect Probe RT-PCR 2X Master Mix, 0.5 µL QuantiTect RT Mix, 0.5 µL Zika1087 (100 µM), 0.5 µL Zika1163c (100 µM), 0.3 µL Zika1108pr-FAM (25 µM) and 5.0 µL RNA from extracted samples. These same samples were evaluated for other flaviviruses by sequencing the amplicons bidirectionally using the Sanger method and comparing the sequence to known flavivirus NS5 sequences in GenBank.

### Network analysis

To evaluate if mosquitoes show specific blood-source selection, or if they feed randomly on available vertebrates, a network analysis was performed. First, an interaction-weighted matrix was built, using mosquito species in rows and vertebrates found through blood source identification in columns. In the matrix, the values used for species interactions corresponded to the number of identified bloodmeals derived from each vertebrate species for each mosquito species. For bipartite network visualization, we used the software GEPHI version 0.9^62^. The network was derived by using the force-directed algorithm FORCEATLAS2 and posterior manual editing. After creating the interaction network, values were calculated for quantitative modularity, complementary specialization and nestedness. Modularity recognizes unexpected species clusters, i.e. species interacting more frequently than expected by chance encounters, based on local availability of vertebrate species. To calculate the modularity, Q, we used the QuanBiMo algorithm with “Beckett” method^63,64^. This algorithm uses quantitative interaction strengths across the community to portray the structure via maximizing weighted modularity. To incorporate stochasticity, we performed 50 iterations with 1,000,000 steps. We selected the command "metaComputeModules" to obtain the maximum possible value and set the modularity calculation. The statistical significance of Q can be assessed with the z-score. A network with a z-score above two is considered significantly modular. Complementary specialization occurs when a mosquito, or group of mosquitoes, feeds selectively on a specific vertebrate species (or group of vertebrates) and is measured using the H2 index. Lastly, the topology of the network at the community level was evaluated with the nestedness metric (NODF index). This index measures the degree of specialist mosquito species interacting with vertebrates that are also used by generalist mosquito species. We calculated the NODF index, with the function "nestednodf" included in the "Vegan" package, following methods previously described^65,66^. A value of 0 indicates non-nestedness, while 100 means complete nestedness^67^.

To identify the ecological traits at vector species level within the network, species specialization, specificity and strength indexes were also calculated. Related to the Shannon diversity index, species specialization (d) reflects the number of vertebrate species utilized for bloodmeals, and ranges from 0 (generalist) to 1 (specialist)^68^. It is calculated with the coefficient of variation of interactions (COV), which is a measure of specificity as described^69^. COV values range between 0 and 1 as minimum and maximum variation, indicating low to high specificity. Lastly, a strength metric (Species Strength) was used to evaluate the species richness (number of vertebrate species) utilized for bloodmeals by each mosquito species^67^, calculated as previously described^70^. To identify the most relevant vertebrate host species within the network, betweenness centrality (BC) was calculated. The BC index describes the importance of a node as a connector between different parts of the network. Nodes with BC > 0 connect areas of the network that would otherwise be sparsely or not connected at all. This BC index has been used to detect potential bridge hosts in other host-parasite networks^40,44^. All the network analyses were performed using the Bipartite package version 2.02 for R version 3.2.

## Supplementary Information

Supplementary Information.

## References

[1] Figueiredo M (2019). Human urban arboviruses can infect wild animals and jump to sylvatic maintenance cycles in South America. Front Cell Infect. Microbiol..

[2] Reeves LE (2018). Interactions between the invasive Burmese python, Python bivittatus Kuhl, and the local mosquito community in Florida. PLoS ONE.

[3] Reeves LE, Gillett-Kaufman JL, Kawahara AY, Kaufman E (2018). Barcoding blood meals: New vertebrate- specific primer sets for assigning taxonomic identities to host DNA from mosquito blood meal. PLoS Negl. Trop. Dis..

[4] Makanga B (2017). “Show me which parasites you carry and I will tell you what you eat”, or how to infer the trophic behavior of hematophagous arthropods feeding on wildlife. Ecol. Evol..

[5] Burkett-Cadena ND, Bingham AM, Porterfield C, Unnasch TR (2014). Innate preference or opportunism: Mosquitoes feeding on birds of prey at the Southeastern raptor center. Vector Ecol..

[6] Mendenhall IH, Tello SA, Neira LA, Castillo LF, Ocampo CB (2012). Host preference of the Arbovirus vector *Culex erraticus* (Diptera: host preference of the arbovirus vector *Culex erraticus* ( Diptera: Culicidae ) at Sonso Lake, Cauca valley department, Colombia. J. Med. Entomol..

[7] Harrington LC (2001). Why do female *Aedes aegypti* (Diptera: Culicidae ) feed preferentially and frequently on human blood?. J. Med. Entomol..

[8] Catenacci LS (2018). Surveillance of Arboviruses in Primates and Sloths in the Atlantic Forest, Surveillance of Arboviruses in Primates and Sloths in the Atlantic Forest, Bahia, Brazil. EcoHealth.

[9] Dos Santos T (2018). Potential of *Aedes albopictus* as a bridge vector for enzootic pathogens at the urban-forest interface in Brazil. Emerging Infect. Dis..

[10] Borremans B (2019). Cross-species pathogen spillover across ecosystem boundaries: mechanisms and theory. Phil. Trans. R. Soc. B..

[11] Weaver SC (2013). Urbanization and geographic expansion of zoonotic arboviral diseases: mechanisms and potential strategies for prevention. Trends in Microbiol..

[12] Caron A, Cappelle J, Cumming GS, Garine-wichatitsky MD, Gaidet N (2015). Bridge hosts, a missing link for disease ecology in multi-host systems. Vet. Res..

[13] Komar N, Clark GG (2006). West Nile virus activity in Latin America and the Caribbean. Rev. Panam. Salud Publica..

[14] Huba Z, Weissenbo H (2010). Zoonotic mosquito-borne flaviviruses: Worldwide presence of agents with proven pathogenicity and potential candidates of future emerging diseases. Vet. Microbiol..

[15] Barrera R, Navarro J, Liria J (2002). Contrasting sylvatic foci of Venezuelan equine encephalitis virus in Northern South America. Am. J. Trop. Med. Hyg..

[16] Hoyos-López R, Soto SU, Rúa-Uribe G, Gallego-Gómez JC (2015). Molecular identification of Saint Louis encephalitis virus genotype IV in Colombia. Mem. Inst. Oswaldo Cruz..

[17] Guzmán C, Calderón A, Martinez C, Oviedo M, Mattar S (2019). Eco-epidemiology of the Venezuelan equine encephalitis virus in bats of Córdoba and Sucre, Colombia. Acta Trop..

[18] Torres-Gutierrez C (2016). Mitochondrial COI gene as a tool in the taxonomy of mosquitoes *Culex* subgenus *melanoconion*. Acta Trop..

[19] Torres-Gutierrez C, Sallum MAM (2015). Catalog of the subgenus *melanoconion* of *Culex* (Diptera: Culicidae) for South America. Zootaxa..

[20] Torres-Gutierrez C, Oliveira TMP, Bergo ES, Sallum MAM (2018). Molecular phylogeny of *Culex* subgenus Melanoconion (Diptera: Culicidae) based on nuclear and mitochondrial protein-coding genes. R Soc Open Sci..

[21] Beebe NW (2018). DNA barcoding mosquitoes: advice for potential prospectors. Parasitol..

[22] Laurito M, De Oliveira TMP, Almirón WR, Anice M, Sallum M (2013). COI barcode versus morphological identification of *Culex* (Culex) (Diptera: Culicidae) species: a case study using samples from Argentina and Brazil. Mem. Inst. Oswaldo Cruz..

[23] IUCN 2020. The IUCN Red List of Threatened Species. https://www.iucnredlist.org (2020).

[24] Roque ALR, Jansen AM (2014). Wild and synanthropic reservoirs of *Leishmania* species in the Americas. Int J Parasitol Parasites Wildl..

[25] Palermo PM (2016). Identification of blood meals from potential Arbovirus mosquito vectors in the peruvian amazon basin. Am. J. Trop. Med. Hyg..

[26] Silva S, Alencar J, Costa JM, Seixas-lorosa E, Guimarães AÉ (2012). Feeding patterns of mosquitoes (Diptera: Culicidae) in six Brazilian environmental preservation areas. J. Vector Ecol..

[27] Edman JD (1971). Host-feeding patterns of florida mosquitoes I. Aedes, anopheles, coquillettidia, Mansonia and Psorophora. J. Med. Entomol..

[28] Gabriel Z (2008). *Culex nigripalpus* Theobald (Diptera, Culicidae) feeding habit at the Parque Ecológico. Rev. Bras. Entomol..

[29] Zimmerman RH, Galardo AK, Lounibos LP, Arruda M, Wirtz R (2006). Bloodmeal Hosts of *Anopheles* Species (Diptera: Culicidae) in a Malaria-Endemic Area of the Brazilian Amazon. J. Med. Entomol..

[30] Mitchell CJ (1987). Hostfeeding patterns of Argentine mosquitoes (Diptera: Culicidae) collected during and after an epizootic of western equine encephalitis. J. Med. Entomol..

[31] Stein M, Zalazar L, Willener JA, Almeida FL, Almirón WR (2013). Culicidae (Diptera ) selection of humans, chickens and rabbits in three different environments in the province of Chaco, Argentina. Mem. Inst. Oswaldo Cruz..

[32] Burkett-Cadena ND (2008). Blood. Feeding patterns of potential arbovirus vectors of the genus. Am. J. Trop. Med. Hyg..

[33] Takken W, Verhulst NO (2013). Host preferences of blood-feeding mosquitoes. Annu. Rev. Entomol..

[34] Besansky NJ, Hill CA, Costantini C (2004). No accounting for taste: host preference in malaria vectors. Trends Parasitol..

[35] Burkett-Cadena ND, Hayes LE (2013). Hosts or habitats: What drives the spatial distribution of mosquitoes? Hosts or habitats. Ecosphere.

[36] Borkent A, Belton P (2006). Attraction of female *Uranotaenia lowii* (Diptera: Culicidae) to frog calls in Costa Rica. Cambridge Univ..

[37] Scott TW, Takken W (2012). Feeding strategies of anthropophilic mosquitoes result in increased risk of pathogen transmission. Trends Parasitol..

[38] Dizney LJ, Ruedas LA (2009). Increased host species diversity and decreased prevalence of Sin nombre virus. Emerg. Infect. Dis..

[39] Krasnov BR (2012). Phylogenetic signal in module composition and species connectivity in compartmentalized host-parasite networks. Am. Nat..

[40] Rodrigues BN, Boscolo D (2020). Do bipartite binary antagonistic and mutualistic networks have different responses to the taxonomic resolution of nodes?. Ecol. Entomol..

[41] Segar S (2020). The role of evolutionary processes in shaping ecological networks. Trends Ecol. Evol..

[42] Svensson-Coelho AM (2014). Reciprocal specialization in multihost malaria parasite communities of birds: A temperate-tropical comparison. Am. Nat..

[43] Ghalmane Z, El Hassouni M, Cherifi C, Cherifi H (2019). Centrality in modular networks. EPJ. Data. Sci..

[44] Rushmore J, Bisanzio D, Gillespie TR (2017). Making new connections: insights from primateparasite networks. Trends Parasitol..

[45] de Carneiro IO (2019). Knowledge, practice and perception of human-marsupial interactions in health promotion. J. Infect. Dev. Ctries..

[46] Root JJ (2005). Serologic evidence of exposure of wild mammals to flaviviruses in the central and eastern United States. Am. J. Trop. Med. Hyg..

[47] Cardoso J (2010). Yellow Fever Virus in *Haemagogus leucocelaenus* and *Aedes serratus*. Emerg. Infect. Dis..

[48] Muñoz M, Navarro JC (2012). Virus Mayaro: un arbovirus reemergente en Venezuela y Latinoamérica. Biomedica.

[49] Turell MJ (2008). Susceptibility of peruvian mosquitoes to eastern equine encephalitis virus. J. Med. Entomol..

[50] Ferro C (2003). Natural enzootic vectors of venezuelan equine encephalitis virus. Emerg. Infect. Dis..

[51] Marsh C, Link A, King-Balley G, Donati G (2016). Effects of fragment and vegetation structure on the population abundance of *Ateles hybridus*, *Alouatta seniculus* and *Cebus albifrons* in Magdalena Valley, Colombia. Folia. Primatol..

[52] Link A, De Luna AG, Alfonso F, Giraldo-Beltran P, Ramirez F (2010). Initial effects of fragmentation on the density of three neotropical primate species in two lowland forests of Colombia. Endanger. Species Res..

[53] Galindo P, Blanton S, Peyton EL (1954). A revision of the *Uranotaenia* of Panama with notes on other American species of the genus (Diptera, Culicidae). Ann. Entomol. Soc. Am..

[54] Forattini, O.P. Culicidologia médica Identificacao, biologia e epidemiologia. 884. (EDUSP, Sao Paulo, 2002).

[55] Folmer, Black M, Hoeh W, Lutz R (1994). 1994 DNA primers for amplification of mitochondrial Cytochrome C oxidase subunit I from diverse metazoan invertebrates DNA primers for amplification of mitochondrial cytochrome c oxidase subunit I from diverse metazoan invertebrates. Mol. Mar. Biol. Biotechnol..

[56] Puillandre N, Lambert A, Brouillet S, Achaz G (2012). ABGD. Automatic barcode gap discovery for primary species delimitation. Mol. Ecol..

[57] Guindon S (2010). New Algorithms and methods to estimate maximum-likelihood phylogenies: Assessing the performance of PhyML 3.0. Syst. Biol..

[58] Molaei G, Andreadis TG, Armstrong PM, Anderson JF, Vossbrinck CR (2006). Host feeding patterns of *Culex* Mosquitoes and West Nile virus transmission, Northeastern United States. Emerg. Infect. Dis..

[59] Ferro C (2011). Phlebotomine vector ecology in the domestic transmission of American cutaneous leishmaniasis in chaparral, Colombia. Am. J. Trop. Med. Hyg..

[60] Moureau G (2007). A real-time RT-PCR method for the universal detection and identification of flaviviruses. Vector Borne Zoonotic Dis..

[61] Lanciotti RS (2008). Genetic and serologic properties of zika virus associated with an epidemic, Yap State. Emerg. Infec. Dis..

[62] Bastian M, Heymann S, Jacomy M (2009). Gephi: an open source software for exploring and manipulating networks. Icwsm..

[63] Beckett SJ (2016). Improved community detection in weighted bipartite networks. R. Soc. Open Sci..

[64] Dormann CF, Strauss R (2014). A method for detecting modules in quantitative bipartite networks. Methods Ecol. Evol..

[65] Almeida-Neto M, Guimara P, Guimara PR, Ulrich W (2008). A consistent metric for nestedness analysis in ecological systems: reconciling concept and measurement. Oikos.

[66] Almeida-Neto M, Ulrich W (2011). Environmental Modelling & Software A straightforward computational approach for measuring nestedness using quantitative matrices. Environ. Model. Softw..

[67] Bascompte J, Olesen JM, Jordano P, Melia CJ (2003). The nested assembly of plant–animal mutualistic networks. PNAS.

[68] Blüthgen N, Menzel F, Blüthgen N (2006). Measuring specialization in species interaction networks. BMC Ecol..

[69] Romain J, Joanne C, Vincent D, Frederic J, Denis C (2006). Spatial segregation of specialists and generalists in bird communities. Ecol. Lett..

[70] Bascompte J, Jordano P, Olesen JM (2006). Facilitate biodiversity maintenance. Science.

