# Supplementary material for: Host selection pattern and flavivirus screening of mosquitoes in a disturbed Colombian rainforest
Source: Sci Rep. 2021 Sep 20;11:18656. doi: 10.1038/s41598-021-98076-8 (PMC8452662; doi:10.1038/s41598-021-98076-8)
Supplement: Supplementary file 1 — Supplementary Information. [file 41598_2021_98076_MOESM1_ESM.docx]

**Host selection pattern and flavivirus screening of mosquitoes in a disturbed Colombian rainforest**

Juliana Hoyos1*, María Cristina Carrasquilla1, Cielo Leon1, Joel M. Montgomery2, Stephanie J. Salyer3, Nicholas Komar4, Camila González1*

^1^ Center for Research in Tropical Microbiology and Parasitology (CIMPAT). Department of Biological Sciences. University of Los Andes, Bogotá. ^2^ Viral Special Pathogens Branch, Division of High Consequence Pathogens and Pathology, National Center for Emerging and Zoonotic Infectious Diseases, U.S. Centers for Disease Control and Prevention, Atlanta, Georgia. ^3^ Global Epidemiology, Laboratory, and Surveillance Branch, Division of Global Health Protection, Center for Global Health, U.S. Centers for Disease Control and Prevention, Atlanta, Georgia. ^4^ Arbovirus Diseases Branch, Division of Vector-Borne Diseases, National Center for Emerging and Zoonotic Infectious Diseases, U.S. Centers for Disease Control and Prevention, Ft. Collins, Colorado.

*Correspondence author: Camila Gonzalez Rosas, Centro de Investigaciones en Microbiología y Parasitología Tropical (CIPAT), Facultad de Ciencias, Departamento de Ciencias Biológicas, Universidad de los Andes, Bogotá – Colombia. E-mail: c.gonzalez2592@uniandes.edu.co

*Correspondence author: Juliana Hoyos, Centro de Investigaciones en Microbiología y Parasitología Tropical (CIPAT), Facultad de Ciencias, Departamento de Ciencias Biológicas, Universidad de los Andes, Bogotá – Colombia. E-mail: mjh58313@uga.edu

Table S1. Vertebrate species recorded by trapping and visual observation within the study site during the sampling period.

| CLASS | Order | FAMILY | GENUS | SPECIES |
| --- | --- | --- | --- | --- |
| AMPHIBIAN | Anura | Bufonidae | *Rinella* | *marina* |
| AMPHIBIAN | Anura | Phyllomedusidae | *Phyllomedusa* | *venusta* |
| AMPHIBIAN | Gymnophiona | Caeciliidae | *Caecilia* | *subnigricans* |
| AMPHIBIAN | Anura | Dendrobatidae | *Dendrobates* | *truncatus* |
| AMPHIBIAN | Anura | Leptodactylidae | *Leptodactylus* | *fuscus* |
| AMPHIBIAN | Anura | Leptodactylidae | *Engystomops* | *pustulatus* |
| AMPHIBIAN | Anura | Microhylidae | *Elachitocleis* | *ovale* |
| AMPHIBIAN | Anura | Hylidae | *Scinax* | sp*.* |
| BIRD | Accipitriformes | Accipitridae | *Rupornis* | *magnirostris* |
| BIRD | Coraciiformes | Alcedinidae | *Chloroceryle* | *americana* |
| BIRD | Coraciiformes | Alcedinidae | *Megaceryle* | *torquata* |
| BIRD | Coraciiformes | Alcedinidae | *Chloroceryle* | *amazona* |
| BIRD | Coraciiformes | Alcedinidae | *Chloroceryle* | *aenea* |
| BIRD | Anseriformes | Anatidae | *Dendrocygna* | *bicolor* |
| BIRD | Anseriformes | Anatidae | *Dendrocygna* | *autumnalis* |
| BIRD | Anseriformes | Anhimidae | *Chauna* | *echavarria* |
| BIRD | Suliformes | Anhingidae | *Anhinga* | *anhinga* |
| BIRD | Pelecaniformes | Ardeidae | *Ardea* | *cocoi* |
| BIRD | Pelecaniformes | Ardeidae | *Bubulcus* | *ibis* |
| BIRD | Pelecaniformes | Ardeidae | *Butorides* | *striata* |
| BIRD | Pelecaniformes | Ardeidae | *Ardea* | *cocoi* |
| BIRD | Pelecaniformes | Ardeidae | *Trigrosoma* | *linneatus* |
| BIRD | Piciformes | Capitonidae | *Notharcus* | *pectoralis* |
| BIRD | Cathartiformes | Cathartidae | *Cathartes* | *aurea* |
| BIRD | Cathartiformes | Cathartidae | *Coragyps* | *Atratus* |
| BIRD | Charadriiformes | Charadridae | *Vanellus* | *chilensis* |
| BIRD | Columbiformes | Columbidae | *Leptotila* | *verreauxi* |
| BIRD | Columbiformes | Columbidae | *Columbina* | *talpacoti* |
| BIRD | Columbiformes | Columbidae | *Patagioenas* | *cayannensis* |
| BIRD | Galliformes | Cracidae | *Ortalis* | *columbiana* |
| BIRD | Cuculiformes | Cuculidae | *Crotophaga* | *ani* |
| BIRD | Cuculiformes | Cuculidae | *Crotophaga* | *sulcirostris* |
| BIRD | Falconiformes | Falconidae | *Milvago* | *chimachima* |
| BIRD | Falconiformes | Falconidae | *Falco* | *sparverius* |
| BIRD | Falconiformes | Falconidae | *Caracara* | *cheriwey* |
| BIRD | Falconiformes | Falconidae | *Herpetotheres* | *chachinans* |
| BIRD | Passeriformes | Furnaridae | *Xiphorhynchus* | *erythropygius* |
| BIRD | Passeriformes | Furnaridae | *Dendroplex* | *picus* |
| BIRD | Galbuliformes | Galbulidae | *Galbula* | *ruficauda* |
| BIRD | Passeriformes | Hirundinidae | *Stelgydopteryx* | *ruficollis* |
| BIRD | Passeriformes | Hirundinidae | *Progne* | *chalybea* |
| BIRD | Passeriformes | Icteridae | *Chrysomus* | *icterocephalus* |
| BIRD | Passeriformes | Icteridae | *Molothrus* | *bonariensis* |
| BIRD | Passeriformes | Icteridae | *Icterus* | *nigrogularis* |
| BIRD | Passeriformes | Icteridae | *Molothrus* | *oryzoborus* |
| BIRD | Passeriformes | incertae sedis | *Saltator* | *maximus* |
| BIRD | Passeriformes | Mimidae | *Mimus* | *gilvus* |
| BIRD | Accipitriformes | Pandionidae | *Pandion* | *haliaetus* |
| BIRD | Suliformes | Phalacrocoracidae | *Phalacrocorax* | *brasilianus* |
| BIRD | Piciformes | Picidae | *Campephilus* | *melanoleuca* |
| BIRD | Piciformes | Picidae | *Melanerpes* | *rubricapillus* |
| BIRD | Piciformes | Picidae | *Dryocopus* | *lineatus* |
| BIRD | Piciformes | Picidae | *Colaptes* | *punctigula* |
| BIRD | Piciformes | Picidae | *Celeus* | *loricatus* |
| BIRD | Passeriformes | Pipridae | *Manacus* | *manacus* |
| BIRD | Psittaciformes | Psittacidae | *Forpus* | *conspicillatus* |
| BIRD | Psittaciformes | Psittacidae | *Amazona* | *ochrocephala* |
| BIRD | Psittaciformes | Psittacidae | *Ara* | *ararauna* |
| BIRD | Psittaciformes | Psittacidae | *Amazona* | *amazonica* |
| BIRD | Psittaciformes | Psittacidae | *Ara* | *severa* |
| BIRD | Piciformes | Ramphastidae | *Pteroglossus* | *torquatus* |
| BIRD | Charadriiformes | Scolopacidae | *Actitis* | *macularius* |
| BIRD | Charadriiformes | Scolopacidae | *Jacana* | *jacana* |
| BIRD | Passeriformes | Thraupidae | *Thraupis* | *episcopus* |
| BIRD | Passeriformes | Thraupidae | *Ramphocelus* | *diamidiatus* |
| BIRD | Passeriformes | Thraupidae | *Sicalis* | *flaveola* |
| BIRD | Passeriformes | Thraupidae | *Thraupis* | *palmarum* |
| BIRD | Passeriformes | Thraupidae | *Euphonia* | *lanirostris* |
| BIRD | Passeriformes | Thraupidae | *Thraupis* | *episcopus* |
| BIRD | Passeriformes | Thraupidae | *Sicalis* | *flaveola* |
| BIRD | Passeriformes | Thraupidae | *Thraupis* | *episcopus* |
| BIRD | Passeriformes | Thraupidae | *Ramphocelus* | *diamidiatus* |
| BIRD | Pelecaniformes | Threskiornithiidae | *Phimosus* | *infuscatus* |
| BIRD | Pelecaniformes | Threskiornithiidae | *Mesenbrinibis* | *cayannensis* |
| BIRD | Pelecaniformes | Threskiornithiidae | *Phimosus* | *infuscatus* |
| BIRD | Tinamiformes | Tinamidae | *Crypturelus* | *soui* |
| BIRD | Apodiformes | Trochilidae | *Phaethornis* | *anthophylus* |
| BIRD | Apodiformes | Trochilidae | *Glaucis* | *hirsutus* |
| BIRD | Passeriformes | Troglodytidae | *Campylorynchus* | *griseus* |
| BIRD | Passeriformes | Tyrannidae | *Pitangus* | *sulfuratus* |
| BIRD | Passeriformes | Tyrannidae | *Schiffornis* | *stenorynchus* |
| BIRD | Passeriformes | Tyrannidae | *Todirostrum* | *cinereum* |
| BIRD | Passeriformes | Tyrannidae | *Tyrannus* | *savana* |
| BIRD | Passeriformes | Tyrannidae | *Myarchus* | *panamensis* |
| BIRD | Passeriformes | Tyrannidae | *Pitangus* | *sulfuratus* |
| BIRD | Passeriformes | Tyrannidae | *Tyrannus* | *melancholicus* |
| BIRD | Passeriformes | Tyrannidae | *Elaenia* | *flavogaster* |
| BIRD | Passeriformes | Tyrannidae | *Rhytipterna* | *holerythra* |
| BIRD | Passeriformes | Tyrannidae | *Fluvicola* | *pica* |
| BIRD | Passeriformes | Tyrannidae | *Megarhynchus* | *pitangua* |
| BIRD | Passeriformes | Tyrannidae | *Myiocetetes* | *cayannensis* |
| BIRD | Passeriformes | Tyrannidae | *Tyrannus* | *melancholicus* |
| BIRD | Passeriformes | Tyrannidae | *Pitangus* | *sulphuratus* |
| BIRD | Passeriformes | Tyrannidae | *Sublegatus* | *areneum* |
| BIRD | Passeriformes | Tyrannidae | *Myarchus* | *pannamensis* |
| BIRD | Passeriformes | Tyrannidae | *Myiocetetes* | *similis* |
| BIRD | Passeriformes | Tytiridae | *Tytira* | *semifasciata* |
| MAMMAL | Chiroptera | Emballonuridae | *Saccopteryx* | *leptura* |
| MAMMAL | Chiroptera | Muridae | *Rattus* | *rattus* |
| MAMMAL | Chiroptera | Phyllostomidae | *Carollia* | *castanea* |
| MAMMAL | Chiroptera | Phyllostomidae | *Platyrrhinus* | *helleri* |
| MAMMAL | Chiroptera | Phyllostomidae | *Carollia* | *perspicillata* |
| MAMMAL | Chiroptera | Vespertilionidae | *Myotis* | *nigricans* |
| MAMMAL | Chiroptera | Phyllostomidae | *Artibeus* | *jamaicensis* |
| MAMMAL | Chiroptera | Phyllostomidae | *Desmodus* | *rotundus* |
| MAMMAL | Chiroptera | Phyllostomidae | *Uroderma* | *bilobatum* |
| MAMMAL | Chiroptera | Phyllostomidae | *Phyllostomus* | *hastatus* |
| MAMMAL | Chiroptera | Phyllostomidae | *Dermanura* | *phaeotis* |
| MAMMAL | Chiroptera | Phyllostomidae | *Glossophaga* | *soricina* |
| MAMMAL | Chiroptera | Thyropteridae | *Thyroptera* | *tricolor* |
| MAMMAL | Chiroptera | Phyllostomidae | *Micronyecteris* | *hirsuta* |
| MAMMAL | Chiroptera | Phyllostomidae | *Lonchophylla* | *thomasi* |
| MAMMAL | Chiroptera | Noctilionidae | *Noctilio* | *albiventris* |
| MAMMAL | Chiroptera | Phyllostomidae | *Gardnerycteris* | *crenulatum* |
| MAMMAL | Chiroptera | Phyllostomidae | *Chrotopterus* | *auritus* |
| MAMMAL | Primates | Atelidae | *Alouatta* | *seniculus* |
| MAMMAL | Chiroptera | Noctilionidae | *Noctilio* | *leporinus* |
| MAMMAL | Rodentia | Echimyidae | *Proechimys* | *chrysaeolus* |
| MAMMAL | Didelphimorphia | Didelphidae | *Didelphis* | *marsupialis* |
| MAMMAL | Chiroptera | Phyllostomidae | *Sturnira* | *lilium* |
| REPTILE | Crocodilia | Alligatoridae | *Caiman* | *crocodilus* |
| REPTILE | Squamata | Boidae | *Corallus* | *ruschenbergerii* |
| REPTILE | Squamata | Colubridae | *Chironius* | *spixi* |
| REPTILE | Squamata | Colubridae | *Leptophis* | *ahaetulla* |
| REPTILE | Squamata | Colubridae | *Pseudoboa* | *neuwiedii* |
| REPTILE | Squamata | Corytophanidae | *Basiliscus* | *basiliscus* |
| REPTILE | Testudines | Emydidae | *Rhinoclemmys* | *melanosterna* |
| REPTILE | Squamata | Iguanidae | *Iguana* | *iguana* |
| REPTILE | Squamata | Teiidae | *Tupinambis* | *teguxin* |
